# Longitudinal Changes in Subfoveal Choroidal Thickness After Phacoemulsification and the Effect of Topical Nepafenac 0.3%

**DOI:** 10.7759/cureus.117523

**Published:** 2026-10-06

**Authors:** Saroj Gupta, Anshukita Anshukita, Priti Singh, Samendra Karkhur, Vidhya Verma, Pragyil Chaturvedi, Vismaya K V

**Affiliations:** 1 Ophthalmology, All India Institute of Medical Sciences, Bhopal, IND; 2 Ophthalmology (Uvea and Anterior Segment Consultancy), Sankara Eye Hospital, Samarjhola, IND

**Keywords:** cataract surgery, edi-oct, nepafenac, nsaid, phacoemulsification, sub-foveal choroidal thickness

## Abstract

Purpose

This study aimed to evaluate longitudinal changes in subfoveal choroidal thickness (SFCT) after uncomplicated phacoemulsification and to assess whether topical nepafenac 0.3% modifies the postoperative SFCT response.

Methods

This prospective observational comparative study included 30 eyes of 30 patients: 15 received standard postoperative therapy (Group A), and 15 received standard therapy plus nepafenac 0.3% once daily (Group B). Postoperative medication was determined independently by the operating surgeon according to routine clinical practice and was not assigned by the investigators for research purposes. SFCT was measured preoperatively and at postoperative day 1 (POD1), week 1, and week 12 using enhanced-depth imaging optical coherence tomography (EDI-OCT). The primary longitudinal analysis used a linear mixed-effects model with patient-level random intercepts and fixed effects for treatment group, time, group-by-time interaction, age, sex, and axial length. Secondary analyses compared changes from baseline with 95% confidence intervals (CIs).

Results

Mean preoperative SFCT was 207.8 ± 18.5 µm in Group A and 194.3 ± 18.9 µm in Group B. SFCT increased in both groups, peaking at week 1 (245.3 ± 14.6 µm and 232.7 ± 21.1 µm, respectively) and declining partially by week 12 (231.5 ± 17.6 µm and 215.0 ± 22.1 µm). SFCT demonstrated a highly significant longitudinal change following phacoemulsification (Wald χ²=97.0; p<0.001). However, the group-by-time interaction was not significant (Wald χ²=0.65; p=0.884). Between-group differences in change from baseline (Group B minus Group A) were −2.07 µm at POD1 (95% CI, −8.97 to 4.84; p=0.542), 0.87 µm at week 1 (95% CI, −12.61 to 14.35; p=0.896), and −3.07 µm at week 12 (95% CI, −13.75 to 7.62; p=0.559).

Conclusion

Phacoemulsification produced a significant longitudinal increase in SFCT, with peak thickening at one week and persistent elevation above baseline at 12 weeks. In this cohort, addition of topical nepafenac 0.3% once daily did not detect any statistically significant difference in the longitudinal SFCT response compared with standard postoperative therapy alone. Larger, adequately powered randomized studies are required to determine whether a clinically meaningful treatment effect exists.

## Introduction

Phacoemulsification has become one of the most commonly performed surgical procedures in ophthalmology worldwide. Cataract remains a major cause of visual impairment, and optimization of postoperative outcomes is particularly important in populations with a high cataract burden [1,2]. Although contemporary phacoemulsification is generally safe and minimally invasive, the procedure can induce measurable changes in posterior-segment tissues that may have clinical relevance.

Cataract surgery can affect the posterior segment through multiple mechanisms. Surgical manipulation and disruption of the blood-aqueous barrier promote the release of pro-inflammatory mediators, including cytokines, leukotrienes, prostaglandins, and growth factors. This postoperative inflammatory cascade may be accompanied by alterations in macular, retinal, and choroidal thickness [3,4]. Because the choroid is highly vascular and dynamically responsive to inflammatory and hemodynamic influences, postoperative changes in choroidal thickness provide a potential structural marker of the ocular response to surgery.

The choroid is the middle vascular layer of the eye and the posterior component of the uveal tract, situated between the retinal pigment epithelium (RPE) and sclera. It is one of the most vascularized tissues in the human body. Choroidal thickness varies with age, sex, axial length, refractive status, and circadian rhythm [5,6]. Histologically, the choroid comprises the suprachoroidal layer, Haller layer, Sattler layer, choriocapillaris, and Bruch membrane [7,8]. Earlier modalities, such as B-scan ultrasonography and indocyanine green angiography, provided indirect assessment of the choroid but limited cross-sectional structural information [9,10]. The enhanced depth imaging (EDI) technique described for spectral-domain optical coherence tomography permits high-resolution visualization of the choroid and sclero-choroidal interface [11]. Subfoveal choroidal thickness (SFCT) is measured perpendicularly from the outer border of the hyperreflective RPE-Bruch membrane complex to the inner scleral boundary beneath the fovea. The reported normative SFCT values vary considerably across populations, pointing up the importance of population-specific interpretation [12].

Topical non-steroidal anti-inflammatory drugs (NSAIDs) have a well-established role in postoperative cataract management [13]. Nepafenac is a prodrug that penetrates ocular tissues and is converted to amfenac, which inhibits cyclooxygenase-based prostaglandin synthesis, reducing inflammation [14]. The posterior-segment reach of topical nepafenac has also been demonstrated experimentally [15]. Previous clinical work has explored whether nepafenac can attenuate postoperative choroidal thickening, although available literature and evidence are limited and have largely involved the 0.1% formulation administered thrice daily [16]. The once-daily 0.3% formulation is simpler for patients to use consistently, but its effect on postoperative SFCT remains insufficiently characterized [17].

The present study therefore evaluated the longitudinal trajectory of SFCT after uncomplicated phacoemulsification with foldable intraocular lens implantation and assessed whether the addition of topical nepafenac 0.3% once daily was associated with a different magnitude of postoperative SFCT change. The analysis also accounted for important baseline determinants of choroidal thickness, particularly axial length.

## Materials and methods

Study design and setting

This was a hospital-based, prospective, observational comparative study conducted in the Department of Ophthalmology, All India Institute of Medical Sciences, Bhopal, between March 2020 and December 2022. The study was conducted in accordance with the Declaration of Helsinki, received Institutional Human Ethics Committee approval (Ref No. IHECPGRMD008, dated 11 March 2020), and all participants provided written informed consent.

Study Population and Treatment Groups

The study included 30 eyes of 30 patients undergoing uncomplicated phacoemulsification with intraocular lens implantation. Postoperative pharmacological management was determined independently by the operating surgeon according to routine clinical practice and was not assigned by the investigators for research purposes. Patients were subsequently categorized according to the postoperative regimen received; and so, the treatment groups represent observational exposure groups rather than randomized treatment groups. Group A had 15 eyes receiving standard postoperative therapy consisting of topical moxifloxacin 0.5% and prednisolone acetate 1%, while Group B had 15 eyes receiving the same regimen with the addition of topical nepafenac 0.3% once daily. Although the approved protocol had planned enrollment of 60 participants, recruitment was substantially curtailed due to the COVID-19 pandemic, resulting in an achieved and analyzable cohort of 30 patients. All statistical analyses, tables, and figures were based on these 30 eyes.

Eligibility Criteria

Inclusion criteria were nuclear sclerosis cataract undergoing uncomplicated phacoemulsification under similar conditions, absence of major systemic illness specified in the protocol, and written informed consent. Exclusion criteria included dense cataract precluding adequate enhanced-depth imaging optical coherence tomography (EDI-OCT) acquisition or OCT signal strength <3, media opacity preventing scanning, uveitis or retinal vascular disease, age-related macular degeneration, corneal pathology, complicated or traumatic cataract, anti-glaucoma medication use, prior ocular surgery or trauma, high myopia (≥6 D), or axial length ≥25 mm.

Clinical assessment and SFCT measurement

Preoperative evaluation included demographic characteristics, best-corrected visual acuity (BCVA), intraocular pressure, slit-lamp biomicroscopy, fundus examination, keratometry, and axial-length measurement. EDI-OCT centered on the fovea was performed using the Cirrus HD OCT-500 (Carl Zeiss Meditec, Dublin, CA, USA). SFCT was measured manually as the perpendicular distance from the posterior border of the hyper-reflective RPE-Bruch membrane complex to the sclero-choroidal junction [11]. Measurements were performed by a single trained observer between 10:00 AM and 11:00 AM to minimize observer and diurnal variability. Measurements were obtained preoperatively and at postoperative day 1 (POD1), week 1, and week 12.

Statistical analysis

Continuous variables are summarized as mean ± standard deviation (SD), and categorical variables as number (percentage). Baseline continuous variables were compared using Welch’s t-test when approximately normally distributed [18] and the Mann-Whitney U test when distributional assumptions were not met; categorical variables were compared using Fisher’s exact test. Because SFCT was measured repeatedly in the same patient, the primary analysis used a linear mixed-effects model with a patient-level random intercept [19]. Fixed effects included group, time, group-by-time interaction, age, sex, and axial length. The group-by-time interaction was the principal test of whether nepafenac modified the postoperative SFCT trajectory. Secondary analyses calculated within-group changes from baseline and between-group differences in change, with 95% confidence intervals (CIs) using the Welch method [18]. For graphical presentation of the mean SFCT at each study time point by treatment group, pointwise 95% CIs around each group mean at each time point were calculated as $\mathrm{Mean} \pm t_{(0.975,\,n-1)} \times \frac{\mathrm{SD}}{\sqrt{n}}$. Exploratory postoperative analysis of covariance (ANCOVA) models adjusted each postoperative SFCT measurement for baseline SFCT, age, sex, and axial length using HC3 heteroskedasticity-consistent robust standard errors [20]; Holm correction was applied across the three postoperative comparisons [21]. All tests were two-sided, and p<0.05 was considered statistically significant. Given the limited sample size, secondary and adjusted analyses were interpreted as exploratory.

## Results

Participant characteristics

The study included 30 eyes of 30 patients, with 15 eyes in each treatment group. The mean age was 64.1 ± 9.6 years in Group A and 58.1 ± 8.1 years in Group B (p=0.074). Group A included 7 males (46.7%) and Group B 5 males (33.3%; Fisher p=0.710). The mean axial length was significantly longer in Group B (23.52 ± 0.49 mm) than in Group A (22.97 ± 0.62 mm; p=0.012). Preoperative SFCT was numerically lower in Group B (194.3 ± 18.9 µm) than Group A (207.8 ± 18.5 µm), although the non-parametric baseline comparison did not reach statistical significance (p=0.085). Baseline characteristics are summarized in Table 1.

**Table 1:** Baseline characteristics of the analyzed cohort Data are mean ± SD unless otherwise specified. Welch’s t-test was used for age and axial length; Mann-Whitney U-test for preoperative BCVA and preoperative SFCT; Fisher’s exact test for categorical variables. BCVA: best-corrected visual acuity; SFCT: subfoveal choroidal thickness

Characteristic	Group A (n=15)	Group B (n=15)	p value
Age, years	64.1 ± 9.6	58.1 ± 8.1	0.074
Male sex, n (%)	7 (46.7)	5 (33.3)	0.710
Right eye, n (%)	9 (60.0)	6 (40.0)	0.462
Preoperative BCVA, logMAR	0.471 ± 0.182	0.517 ± 0.155	0.368
Preoperative SFCT, µm	207.8 ± 18.5	194.3 ± 18.9	0.085
Axial length, mm	22.97 ± 0.62	23.52 ± 0.49	0.012

Longitudinal SFCT changes

SFCT increased after surgery in both groups, with the highest mean values at week 1. In Group A, the mean SFCT increased from 207.8 ± 18.5 µm preoperatively to 224.1 ± 20.3 µm at POD1, 245.3 ± 14.6 µm at week 1, and 231.5 ± 17.6 µm at week 12. In Group B, corresponding values were 194.3 ± 18.9 µm, 208.6 ± 19.5 µm, 232.7 ± 21.1 µm, and 215.0 ± 22.1 µm. Figure 1 shows the longitudinal means with 95% CIs.

**Figure 1:**
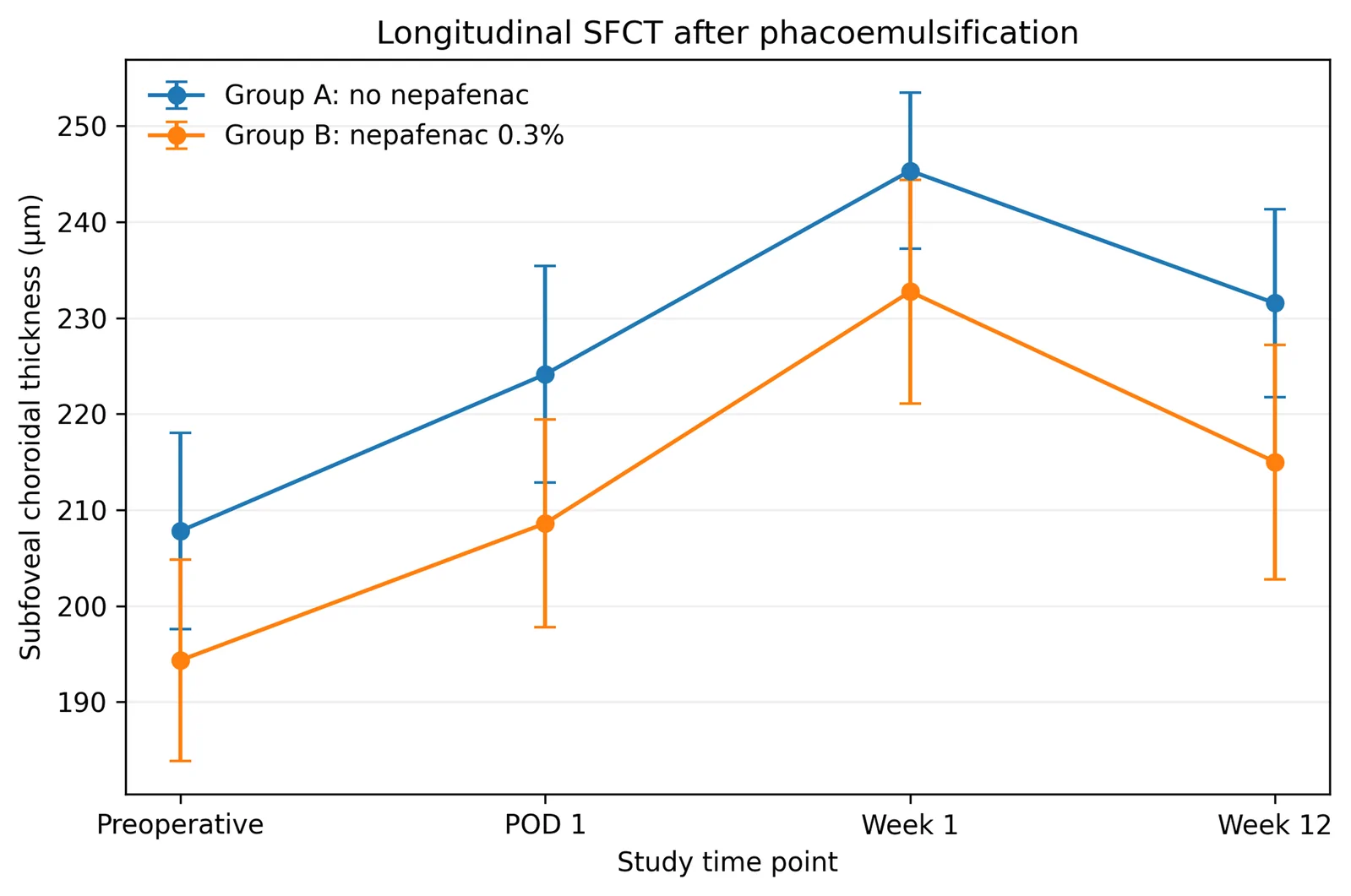
Mean subfoveal choroidal thickness at each study time point by treatment group Error bars represent pointwise 95% confidence intervals calculated from the group-specific mean and standard error at each time point.

The mixed-effects model demonstrated a highly significant longitudinal effect of time (Wald χ²=97.0; p<0.001), confirming substantial postoperative change in SFCT, with peak mean thickening at week 1. However, the overall group-by-time interaction was not significant (Wald χ²=0.65; p=0.884), indicating no evidence that the postoperative SFCT trajectory differed between groups after accounting for age, sex, and axial length.

Change-from-baseline comparisons

Mean SFCT change at POD1 was +16.33 ± 10.88 µm in Group A and +14.27 ± 7.03 µm in Group B. At week 1, changes were +37.53 ± 14.36 µm and +38.40 ± 20.88 µm, respectively; at week 12, changes were +23.73 ± 10.85 µm and +20.67 ± 16.86 µm. None of the between-group differences in change was statistically significant: −2.07 µm at POD1 (95% CI, −8.97 to 4.84; p=0.542), +0.87 µm at week 1 (95% CI, −12.61 to 14.35; p=0.896), and −3.07 µm at week 12 (95% CI, −13.75 to 7.62; p=0.559). These estimates are displayed in Figure 2 and Table 2.

**Figure 2:**
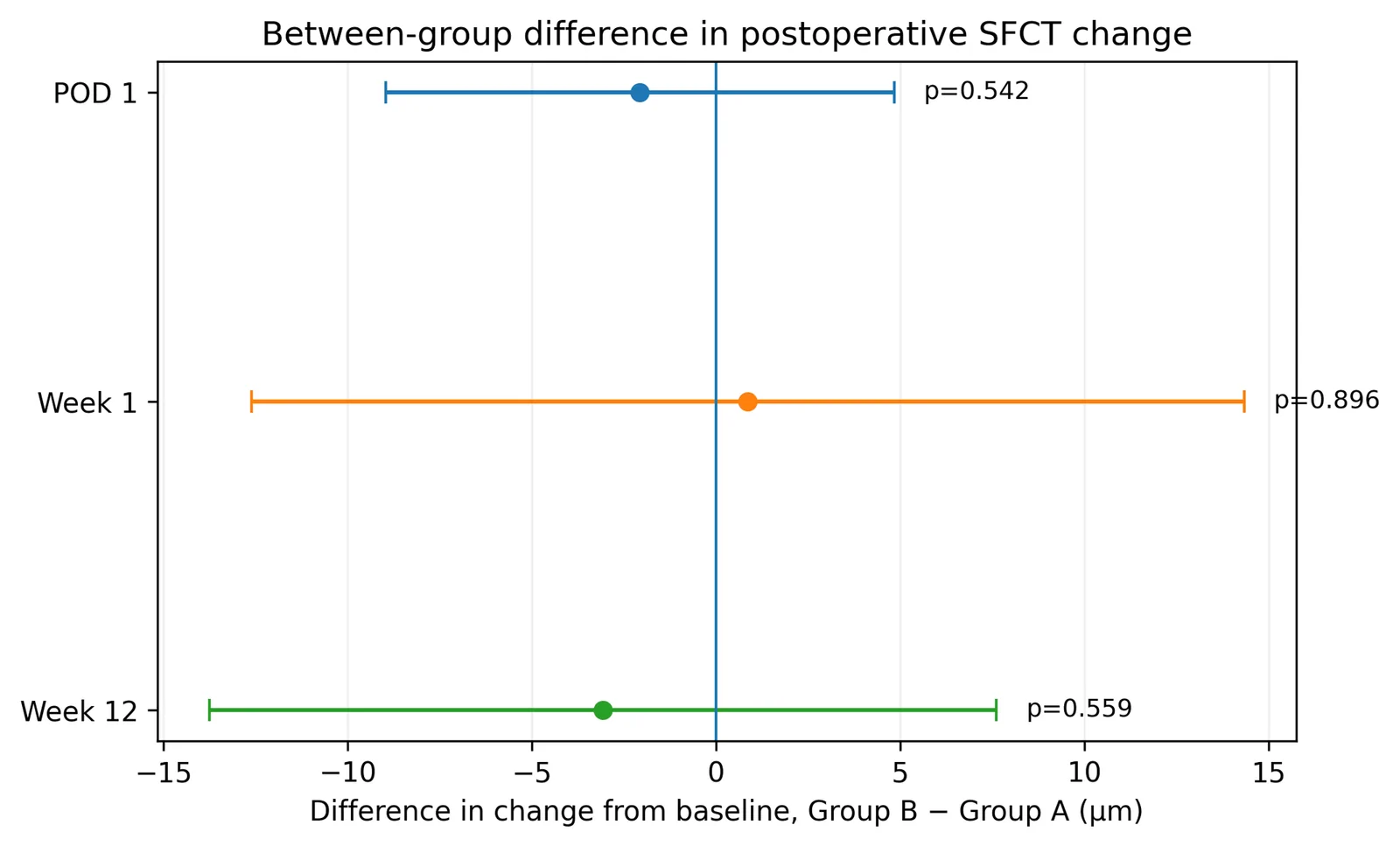
Forest plot of between-group differences in SFCT change from baseline (Group B minus Group A) Negative values favor a smaller increase in the nepafenac group. Error bars represent 95% confidence intervals. SFCT: subfoveal choroidal thickness

**Table 2:** Longitudinal SFCT and change from baseline SFCT values and within-group changes are mean ± SD. The between-group effect is Group B minus Group A change from baseline with 95% CI; p values are Welch comparisons of change scores. SFCT: subfoveal choroidal thickness

Time point	Group A SFCT, µm	Group B SFCT, µm	Change A, µm	Change B, µm	B−A change (95% CI)	p value
Preoperative	207.80 ± 18.50	194.33 ± 18.92	—	—	—	—
POD1	224.13 ± 20.35	208.60 ± 19.52	16.33 ± 10.88	14.27 ± 7.03	−2.07 (−8.97 to 4.84)	0.542
Week 1	245.33 ± 14.65	232.73 ± 21.06	37.53 ± 14.36	38.40 ± 20.88	0.87 (−12.61 to 14.35)	0.896
Week 12	231.53 ± 17.62	215.00 ± 22.06	23.73 ± 10.85	20.67 ± 16.86	−3.07 (−13.75 to 7.62)	0.559

Exploratory adjusted postoperative comparisons

In exploratory ANCOVA models adjusting for baseline SFCT, age, sex, and axial length, the adjusted Group B minus Group A difference was −3.31 µm at POD1 (95% CI, −12.78 to 6.15; p=0.493), −7.32 µm at week 1 (95% CI, −19.27 to 4.63; p=0.230), and −11.17 µm at week 12 (95% CI, −21.75 to −0.59; nominal p=0.038). After Holm correction for the three postoperative comparisons, the week-12 finding was no longer statistically significant (adjusted p=0.115). These exploratory adjusted estimates are summarized in Table 3.

**Table 3:** Primary longitudinal model and exploratory adjusted postoperative comparisons Mixed-effects model included a patient-level random intercept and fixed effects for group, time, group×time, age, sex, and axial length. Exploratory ANCOVA models adjusted postoperative SFCT for baseline SFCT, age, sex, and axial length with HC3 robust standard errors. Holm correction was applied across the three postoperative ANCOVA comparisons. SFCT: subfoveal choroidal thickness; ANCOVA: analysis of covariance

Analysis	Estimate/test	95% CI	p value	Multiplicity-adjusted p
Mixed model: overall time effect	Wald χ²=97.00 (3 df)	—	<0.001	—
Mixed model: group × time interaction	Wald χ²=0.65 (3 df)	—	0.884	—
Adjusted Group B−A at POD1	−3.31 µm	−12.78 to 6.15	0.493	0.493
Adjusted Group B−A at Week 1	−7.32 µm	−19.27 to 4.63	0.230	0.460
Adjusted Group B−A at Week 12	−11.17 µm	−21.75 to −0.59	0.038	0.115

## Discussion

This prospective comparative analysis demonstrates a clear postoperative increase in SFCT after uncomplicated phacoemulsification. In both treatment groups, SFCT increased by POD1, reached its maximum mean value at week 1, and then partially declined by week 12 while remaining above the preoperative level. This temporal pattern is consistent with previous reports by Cevher et al., Pierru et al., Jiang et al., Shahzad et al., and Gudauskiene et al., all of whom documented postoperative choroidal thickening after cataract surgery [16,22-25]. The week-1 peak observed in the present cohort is biologically plausible because it corresponds to the early postoperative phase in which disruption of the blood-aqueous barrier and prostaglandin-mediated inflammatory activity are most pronounced.

The mechanism behind postoperative choroidal thickening is likely multifactorial. Phacoemulsification can initiate the release of prostaglandins and cytokines, increase vascular permeability, and alter posterior-segment blood flow. Surgical ultrasound energy and operative light exposure may provide additional inflammatory or phototoxic stimuli. Together, these processes may produce transient expansion of the choroidal vascular and stromal compartments. The persistence of SFCT above baseline at 12 weeks in both groups suggests that choroidal recovery may extend beyond the period of clinically apparent anterior-segment inflammation.

The principal comparative question was whether postoperative SFCT trajectories differed between the two treatment groups. Although Group B had lower absolute SFCT values at each postoperative time point, the mixed-effects model did not demonstrate a significant group-by-time interaction (p=0.884), and direct between-group comparisons of change from baseline were small and statistically non-significant at POD1, week 1, and week 12. Thus, no statistically significant difference in the longitudinal postoperative SFCT response was detected between the two groups.

This interpretation is important because absolute postoperative thickness alone can be misleading when groups differ at baseline. Group B began with a numerically thinner choroid and had a significantly longer mean axial length (p=0.012). Because longer axial length is generally associated with a thinner choroid [5,25], some of the apparent absolute postoperative difference between groups may reflect pre-existing anatomy rather than a pharmacological effect. This baseline axial-length imbalance therefore represents an important potential confounder when interpreting the between-group SFCT differences. Change-from-baseline comparisons and a repeated-measures model are consequently more appropriate for assessing whether postoperative responses differed between the treatment groups.

The exploratory covariate-adjusted ANCOVA models yielded a lower adjusted SFCT in Group B at week 12, with a nominal p value of 0.038 after adjustment for baseline SFCT, age, sex, and axial length. However, this association did not remain statistically significant after Holm correction for the three postoperative comparisons (adjusted p=0.115). Accordingly, the week-12 finding should not be interpreted as statistically significant evidence of a treatment effect. Given the small sample size and observational treatment allocation, this signal should be interpreted as hypothesis-generating rather than as confirmatory evidence of a delayed nepafenac effect.

Previous studies have examined topical NSAIDs in relation to postoperative posterior-segment inflammation. Cevher et al. evaluated nepafenac 0.1% three times daily and reported a tendency toward lower postoperative choroidal thickness without a consistently significant treatment effect [16]. Tzelikis et al. demonstrated posterior-segment anti-inflammatory efficacy of nepafenac 0.3% in the prevention of postoperative macular edema, supporting the biological plausibility of an effect beyond the anterior segment [17]. Differences among published studies may relate to formulation and dosing, imaging technology, baseline SFCT, axial length, timing of OCT acquisition, surgical technique, and statistical methodology. The once-daily 0.3% formulation remains clinically attractive because of simpler dosing, but its specific effect on choroidal thickness requires confirmation in a larger randomized cohort.

The strengths of the present analysis include individual-level longitudinal measurements, standardized EDI-OCT acquisition timing, measurements by a single trained observer, and a statistical approach that explicitly accounted for repeated observations and major baseline determinants of SFCT. The main limitation is the smaller-than-planned sample of 30 eyes (15 per group); recruitment was substantially curtailed during the COVID-19 pandemic, reducing statistical power and widening CIs. The groups also differed in axial length and showed a numerical baseline SFCT difference, creating potential residual confounding despite adjusted analyses. Manual SFCT measurement introduces measurement variability, and procedures performed by multiple surgeons may have added surgical heterogeneity. Treatment allocation reflected routine postoperative prescribing practices of the operating surgeons and was not assigned by the investigators; therefore, causal inference regarding nepafenac should remain cautious. The small sample means that the present findings should be considered exploratory and should be validated in an adequately powered prospective randomized study.

## Conclusions

Phacoemulsification produced a significant longitudinal increase in SFCT, with the greatest mean thickening at approximately 1 week and persistent elevation above baseline at 12 weeks. No statistically significant difference in the overall SFCT trajectory or change from baseline was detected between patients receiving topical nepafenac 0.3% once daily in addition to standard postoperative therapy and those receiving standard therapy alone. Apparent differences in absolute postoperative SFCT should be interpreted in light of the baseline SFCT and axial-length imbalance between groups. EDI-OCT provides a useful non-invasive method for monitoring postoperative choroidal changes. Larger, adequately powered randomized studies with prespecified longitudinal analyses are required to determine whether nepafenac 0.3% has a clinically meaningful effect on post-phacoemulsification choroidal thickening.
